# P-1397. Efficacy of Novel Tetracyclines versus Best Available Therapy in Treating Mycobacterium Abscessus Pulmonary Infections

**DOI:** 10.1093/ofid/ofaf695.1584

**Published:** 2026-01-11

**Authors:** Mealis Taouk, William L Musick, Shivani Patel, Jiejian Lin, Kevin Grimes

**Affiliations:** Houston Methodist Hospital, houston, Texas; Houston Methodist Hospital, houston, Texas; Houston Methodist, Houston, Texas; Houston Methodist Hospital, houston, Texas; Center for Cell and Gene Therapy, Baylor College of Medicine, Texas Children’s Hospital, Houston Methodist Hospital, Houston, Texas

## Abstract

**Background:**

*Mycobacterium Abscessus* is a rapidly growing pathogen causing severe pulmonary infections, especially in immunocompromised hosts. Treatment is limited by intrinsic drug resistance, few effective agents, and drug induced toxicity. Novel tetracyclines (tigecycline, eravacycline, omadacycline) are increasingly used in clinical practice, though outcomes data remain limited.

**Figure:**
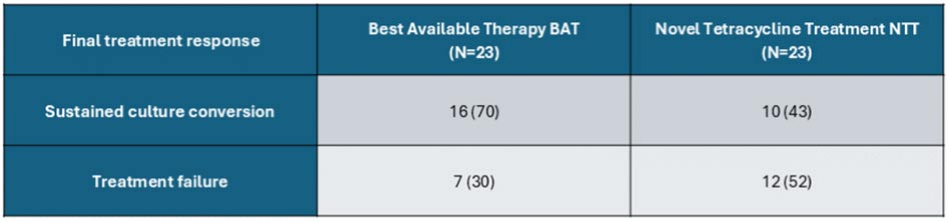
Primary Endpoint - Culture Conversion in NTT and BAT Data are shown as No. (%) unless otherwise indicated

**Figure:**
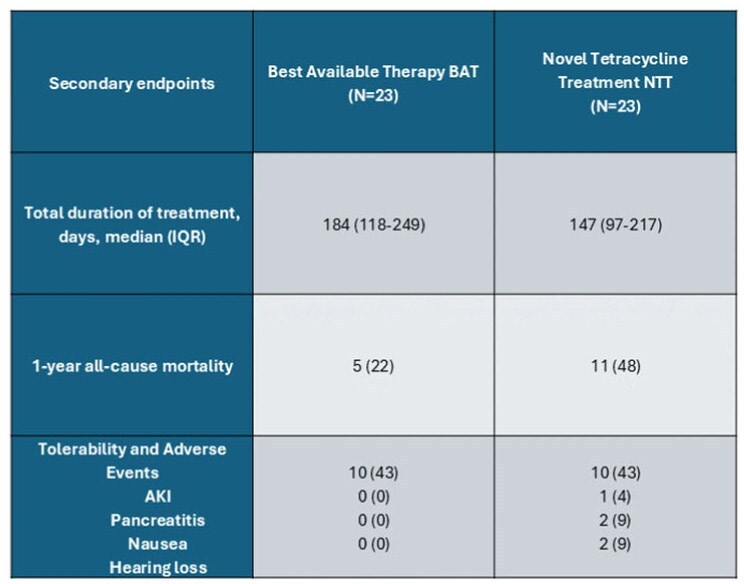
Secondary Endpoints Data are shown as No. (%) unless otherwise indicated. Abbreviations: AKI, Acute Kidney injury

**Methods:**

This retrospective cohort study at Houston Methodist Hospital System compared the efficacy and safety of novel tetracycline-based therapies (NTT) to best available therapy (BAT) for *M. abscessus* pulmonary infections. Adults (≥ 18 years) with culture-confirmed disease treated with ≥ 2 active agents within 30 days of index culture and for ≥ 4 weeks between May 2016–Dec 2023 were included. Those with < 6 months of follow-up were excluded unless death occurred earlier. The NTT group received ≥ 1 tetracycline agent in combination with agents such as macrolides, linezolid, and inhaled amikacin. The BAT group were treated without tetracyclines, typically azithromycin-based regimens with inhaled amikacin, imipenem, and/or linezolid. The primary outcome was microbiological response, defined as sustained culture conversion at 12 months or end of therapy; treatment failure was defined as failure to achieve culture conversion or recurrence of positive cultures after initial conversion. Secondary outcomes included adverse drug events, treatment duration, regimen modifications, in vitro susceptibility, and all-cause mortality at 12 months.

**Results:**

46 patients met the inclusion criteria (n = 23 per group), most immunocompromised. Culture conversion was higher with BAT (70%) vs NTT (43%). BAT had fewer adverse events and lower 12-month mortality (22% vs. 48%). Amikacin susceptibility was >85% in both groups, while NTT had more macrolide resistance, initial AFB smear positivity, and regimen changes. Tigecycline (61%) was the most frequently used tetracycline.

**Conclusion:**

BAT was associated with better microbiologic outcomes and fewer toxicities compared to NTT. Prospective studies are needed to better define the efficacy and safety of novel tetracycline in *M. abscessus* pulmonary infections.

**Disclosures:**

All Authors: No reported disclosures

